# Intensive care physicians’ experiences of decision fatigue and characteristics of vulnerable clinical decisions

**DOI:** 10.1038/s41598-026-57919-y

**Published:** 2026-06-13

**Authors:** Lorenz Schiessl, Anne Herrmann, Richard-Felix Kraus, Viktoria Kimmerling, Johanna Rosenberger, Cynthia Kohl, Martin Georg Kees, Alexander Leonhard Leibold

**Affiliations:** 1 Department of Anaesthesiology, University Hospital of Regensburg, Franz-Josef- Strauss-Alle 11, 93053 Regensburg, Germany; 2 Department of Epidemiology and Preventive Medicine, Medical Sociology, University Hospital of Regensburg, Franz-Josef-Strauss-Alle 11, 93053 Regensburg, Germany; 3 University Department of Obstetrics and Gynaecology, Clinic St. Hedwig of The Order of St. John, University of Regensburg, Steinmetzstr. 1-3, 93049 Regensburg, Germany

**Keywords:** Decision fatigue, Patient safety, Staff wellbeing, Clinical decision making, Intensive care unit, Semi-structured interview, Qualitative research, Health care, Medical research, Psychology, Psychology

## Abstract

Decision fatigue (DF) has been proposed to describe changes in decision-making over the course of repeated decisions, but its mechanisms and relevance in clinical practice remain debated. While quantitative studies have reported time-related patterns in medical decisions, qualitative evidence on how DF is experienced and managed in everyday clinical settings is limited. Intensive care units (ICUs), characterised by high decision density, time pressure, and uncertainty, provide a particularly relevant context to explore these processes. This study explored intensive care physicians’ views and experiences on DF and its potential impact on medical decision making in the ICU. 19 semi-structured interviews were conducted in person with ICU physicians from October 2024 to March 2025. All interviews were audio-recorded, transcribed verbatim, and analysed using an inductive thematic analysis approach. Codes and categories were iteratively developed and grouped into higher-level themes and decision characteristics. 19 physicians from three different ICUs participated, including 13 residents and board-certified specialists in executing roles and 6 consultants with supervisory and treatment-planning responsibilities. Three major themes were identified and developed: (1) DF and mental exhaustion occur in the ICU; (2) Perceived effects of DF on decision-making processes and behaviours; and (3) physicians indicate various characteristics of decisions in which the effects of DF are more likely to occur. This study provides new insights into ICU physicians’ experiences of DF and presents a typology of clinical decisions according to their perceived susceptibility to DF as a hypothesis-generating framework. The findings suggest practical implications for workflow design, decision prioritisation, and team-based approaches to support clinical decision-making in the ICU.

## Background

Making decisions is an essential part of medical practice, whether it involves prescribing medication, evaluating diagnostic information, or performing administrative tasks^1^. A study by Ofstad et al. showed that physicians make an average of 13 clinical decisions per patient contact^2^. Clinical decision-making can be broadly defined as “a contextual, continuous, and evolving process, where data are gathered, interpreted, and evaluated in order to select an evidence-based choice of action.“^3^.

Decision fatigue (DF) is commonly used to describe changes in decision-making performance over the course of repeated decisions. Early theoretical accounts that can be used to explain the phenomenon, such as Roy Baumeister’s strength model of self-regulation, conceptualized this as a depletion of self-regulatory capacity^4^.

More recent perspectives propose alternative explanations^5^. One approach focuses on perceived resource scarcity, which views cognitive strain as arising from the subjective experience of having insufficient mental resources and the resulting tendency to narrow attention and prioritise immediate rather than long-term goals^6^. Another emphasises the opportunity costs of mental effort, conceptualising mental fatigue as an adaptive signal that continued cognitive engagement may provide less overall gain relative to competing uses of attention and effort^7^.

Considering these debates, DF can be understood as a multifactorial and context-dependent phenomenon rather than the result of a single underlying mechanism.

When numerous decisions must be made within a short period, individuals may experience changes in how decisions are approached, potentially leading to a gradual decline in consistency or effort invested in decision-making. As work periods lengthen, individuals tend to rely more heavily on cognitive shortcuts^8^, for example by defaulting to routine or conservative options, postponing decisions, or selecting less effortful courses of.

Research outside medicine illustrates similar patterns. In a well-known study, Danziger et al. found that judges became increasingly restrictive in their probation decisions as the working day progressed^9^. In another study, credit officers showed a decline in loan approvals during midday and late afternoon hours^10^. These findings suggest that DF may influence decision-making across various professional domains. Comparable trends have been observed in clinical work. Studies have demonstrated an increase in the prescription of opioids^11^ and antibiotics for acute respiratory infections^12^ over the course of the day, and surgeons were less likely to perform surgical procedures toward the end of a shift^13^. These examples are consistent with potential fatigue-related shifts in clinical decision-making.

While these findings show consistent patterns of change in decision-making over time, they do not establish DF as the underlying causal mechanism. Such effects could also reflect other influences, including time pressure, workflow dynamics, or patient-related factors. Therefore, they should be interpreted with caution.

However, not all studies have identified such effects. A recent review found that, among 82 included studies, only 45% of those quantitatively assessing DF reported significant associations with diagnoses, test ordering, prescribing, or therapeutic decisions^14^. With only one qualitative study identified, the review highlighted a lack of qualitative evidence, limiting insight into how DF is perceived and managed in everyday clinical practice. The authors concluded that qualitative approaches are needed to provide a more nuanced understanding of DF and to inform the development of targeted interventions^14^.

Given the ongoing debate about the mechanisms of DF and the limited qualitative evidence available, a deeper understanding is needed of how clinicians experience and interpret decision-making under conditions of high demand and limited resources. Intensive care units (ICUs) provide a particularly relevant context in this regard: they involve many complex, high-stakes decisions made under uncertainty, time pressure, frequent interruptions, and emotional strain. Many of these decisions must be made simultaneously, requiring constant prioritisation and trade-offs between competing demands. These conditions increase cognitive load and may shape how decision processes evolve over time.

Therefore, we conducted interviews with 19 intensivists to explore their subjective experiences of DF, to identify decision characteristics that may render clinical decisions more or less susceptible to its effects, and to derive potential strategies for its prevention and mitigation.

## Methods

### Study design

A qualitative study design using semi-structured interviews was chosen to gain in-depth insights into physicians’ experiences with DF. The interview guide based on an extensive literature review and was developed by an interdisciplinary research team involving physicians and health service researchers. It was pilot tested with two intensive care physicians who were not included in the final sample, and minor adjustments were made prior to data collection. The guide was iteratively refined throughout the interview process to incorporate emerging insights from the data.

### Participants and recruitment

The Ethics Committee of the University of Regensburg issued a declaration of non-objection, confirming that formal ethical approval was not required in accordance with § 15 of the Professional Code of Conduct for Physicians in Bavaria. All methods were performed in accordance with relevant guidelines and regulations and in accordance with the Declaration of Helsinki. Recruitment was carried out through purposive sampling^15^ among intensivists of our Department of Anaesthesiology, which runs three surgical ICUs. In order to capture a broad spectrum of different levels of clinical practice and corresponding decision-making experiences and perspectives, junior doctors within their first year of intensive care training, board certified anaesthetists during their specialization in intensive care and finally senior specialists with several years of specialization were invited. Recruitment was conducted via email by A.L.L., who had no supervisory relationship with any of the participants. A total of 25 physicians were invited to take part, comprising 23 clinicians routinely staffing the department’s three ICUs and 2 in senior leadership roles in intensive care medicine. Of these, 19 physicians participated, none declined, and 6 did not respond. Reasons for non-participation were not systematically recorded. No incentives were provided. Participants received written and oral study information by the research team. All participants were adults and were provided written informed consent for the participation and the publication of pseudonymised information prior to participation. Nineteen physicians participated in the study, and their demographic characteristics are presented in Table 1.

### Data collection

The interviews were conducted in person by L.S., a medical student in the fourth year of medical school, between October 2024 and March 2025. L.S. had no supervisory or hierarchical relationship with any of the participants. Prior to the study, L.S. received training in qualitative research methods and interview techniques from A.H. To minimise potential influence on participants’ responses, interviews were conducted in a confidential setting during regular working hours in a meeting room at the respective ward. Participants were explicitly informed that participation or non-participation would have no impact on their professional evaluation or working conditions.

All interviews were audio recorded using Audacity^16^ and pseudonymised before being transcribed verbatim using the transcription function of Microsoft Word 365^17^ and were checked against the original audio recordings and manually corrected where necessary to ensure accuracy.

### Data analysis

An inductive thematic analysis approach was used to evaluate the interview data. Transcripts were read multiple times to familiarise with the data. Subsequently, meaning units were identified, openly coded, and systematically categorised. The first three transcripts were independently coded by two researchers (L.S. and A.L.L.) to ensure the reliability and consistency of the coding process. Any differences in coding were discussed and resolved by consensus. Data analysis was supported by MAXQDA software^18^, enabling structured organization and management of the data. As the analysis progressed, codes were continuously compared, refined, and grouped into higher-level categories. Particular attention was given to ambiguous and borderline cases especially in the distinction of different decision types. A.H. provided ongoing supervision and methodological reflection throughout the entire process. Analysis continued until no new relevant concepts emerged in consecutive interviews^15^. This approach was informed by the concept of information power, which considers sample adequacy in relation to study aim, sample specificity, and data quality^19^. This assessment was conducted iteratively during the analysis process by L.S. and A.L.L. and was discussed within the research team to ensure consensus. Interviews were conducted in German and quotations were translated into English by the research team. Translations were reviewed to preserve meaning and ensure readability by A.L.L., who is bilingual in German and English.

**Table 1:** Demographic data for participants.

Participant	Clinical role	Experience working on ICU (years)	Gender
P1	Consultant	1–5	Male
P2	Consultant	> 5	Male
P3	Consultant	> 5	Male
P4	Consultant	> 5	Male
P5	Consultant	> 5	Male
P6	Consultant	> 5	Male
P7	Resident	1–5	Male
P8	Resident	1–5	Male
P9	Resident	≤ 0.5	Male
P10	Resident	1–5	Male
P11	Resident	≤ 0.5	Female
P12	Resident	1–5	Male
P13	Resident	1–5	Female
P14	Resident	1–5	Female
P15	Resident	0.5–1	Male
P16	Resident	0.5–1	Female
P17	Resident	1–5	Female
P18	Resident	≤ 0.5	Female
P19	Resident	0.5-1	Female

## Results

The interviewees comprised 13 residents and board-certified specialists in executing roles, and 6 consultants with supervisory and treatment-planning responsibilities from three different ICUs of a university hospital. The length of the interviews varied from 22 to 57 min and the IQR was 30.5 min. Analysis of the data revealed three major themes, which are described in detail below.

### Experiences of mental exhaustion and DF in ICU practice

Most participants reported experiencing mental or physical exhaustion during their shifts, closely linked to the *sheer number of decisions* (P12) made. Several described a gradual depletion of *cognitive energy* (P15) over the course of a shift, particularly on days with dense sequences of clinical choices. The accumulation of numerous small and seemingly inconsequential decisions, such as whether a patient may take a sip of water, was perceived as unexpectedly draining and often more exhausting than a small number of major decisions, such as whether a patient should be intubated. Participants described DF as manifesting as a narrowing of mental bandwidth, with routine tasks requiring greater effort and previously straightforward decisions becoming disproportionately effortful. This was commonly attributed to a perceived reduction of attentional and working-memory resources, which they felt reduced the capacity to process information efficiently and was associated with an increased reliance on low-effort cognitive strategies. The threshold at which these effects became noticeable varied substantially between individuals. Rather than a clearly defined moment, participants described a gradual, often subtle onset that became apparent only once they felt mentally slowed or disproportionately strained by routine tasks. Participants reported that situational factors such as stable staffing levels, predictable workflows, or uninterrupted breaks could delay the effects` perceived onset, whereas poor physical condition, accumulated stress, or limited clinical experience tended to accelerate it.

Although most participants highlighted a link between decision load and fatigue, a few indicated that routine decisions *should not impact fatigue or decision quality* (P7), reflecting an ideal of cognitive efficiency. However, even they acknowledged that personal circumstances or external pressures occasionally reduced their capacity to handle decision-dense shifts.

I think I have a certain number of decisions I can make per day and when I reach a certain moment, I just can’t go on anymore. (P18)

The more of these small decisions there are, the more tiring it is, the less motivated you are, the more you want to conserve yourself and avoid this irrelevant petty crap [laughs]. (P8)

### Decision-making as a core component of professional identity

Many participants described medical decision-making and the responsibility associated with it as central to their professional identity. Making decisions was not only perceived as an operational necessity but as a meaningful and defining aspect of their role as physicians.

It is my task to make decisions, to bring patients forward. A day without decisions feels like a day lost. (P5)

I also have the expectation of myself, that I become more independent and am able to act on my own. That’s why I try to make as many decisions myself as possible. (P9)

### Perceived effects of DF on decision-making processes and behaviours

Most participants reported that DF was perceived to affect several cognitive and behavioural aspects of decision-making. Concentration commonly declined under high decision load, described as *slowing down*,* not considering certain aspects* (P9), with earlier decisions being revised more frequently (P14). Many described increased reliance on heuristics: *We’ve been doing it this way recently*,* so it’s probably fine. (P11*), framed as low-effort strategies to conserve limited cognitive resources.

Many participants also perceived DF as increasing their vulnerability to errors. They described re-reading documentation without processing it (P11) and making procedural inaccuracies - especially in medication prescription - despite best intentions, which they attributed to diminished *cognitive capacity rather than negligence* (P15). Some reported becoming *more impulsive*,* less deliberate*,* more driven by gut feeling* (P8), while others reported the opposite reaction: avoidance, delay, or passing decisions to the next shift. Increased irritability, reduced empathy, and a decline in communication quality were also reported by several participants. In particular, participants described that explanations to colleagues, patients and relatives became shorter, less nuanced, and less patient, which occasionally resulted in misunderstanding or dissatisfaction. Physical symptoms such as headaches, tension and general exhaustion were also described in this context.

Sometimes in the evening, you sit in front of your computer, and you read the same sentence over and over again without even getting what it says. (P11)

Errors happen, you are not as attentive when prescribing meds, well you are putting in effort but you’re just not that capable anymore and make hasty mistakes you wouldn’t if you were rested. (P15)

I definitely tend to pass the decision on to someone else. That’s just the way it is, to be honest, everyone has to admit that, because I think we all do that to some extent. (P13)

Yes, you’re definitely more impulsive, less thoughtful and you make more gut decisions, yes. (P8)

### Physicians indicate various characteristics of decisions in which the effects of DF are more likely to occur

Results indicate that not all decisions were equally affected by DF. Participants identified specific decisional characteristics that either protected against or amplified the effects of DF.

### Decision characteristics associated with lower susceptibility to DF

Emergency situations were consistently described as relatively resistant to DF. Even when fatigued, physicians reported being *able to draw on professionalism and remaining concentration* (P7) to act effectively. The presence of well-rehearsed algorithms provided some degree of cognitive scaffolding: *Emergencies follow fixed routines… I can do that even when I’m exhausted because it’s automatic* (P11; P14). Physicians emphasised that in emergencies, action is obligatory despite uncertainty: *You might make a wrong decision but doing nothing isn’t an option* (P11). They noted that while DF could emerge afterwards, it generally was perceived to have limited influence during acute decision-making.

Participants described ethically weighty or high-consequence decisions - such as withdrawing life-sustaining treatment, escalating treatment, or determining key diagnostic steps - as less affected by DF. These decisions were typically made collaboratively within a multidisciplinary team (P3, P6), which participants perceived as reducing the influence of individual fatigue. Furthermore, such decisions often evolved over days rather than minutes, allowing clinicians to verify information, revisit judgements with a rested mind, and distribute cognitive responsibility across the team.

Depending on how difficult the decision is, especially when it comes to tough, serious decisions - I’d rather double-check, verify, and then make the decision. (P7)

[When making decisions with serious consequences, it’s important] that they are distributed and well justified. To really weigh everything again and discuss all the alternatives again, because once the decision is made, there’s no turning back. (P8)

### Decision characteristics associated with higher susceptibility to DF

In contrast, participants highlighted that certain characteristics make decisions more susceptible to the effects of DF described above. These characteristics are outlined in the following sections.

### High anticipated effort or workload

Physicians reported that decisions leading to physical action were more difficult to initiate when fatigued. For example, going to the bedside for a clinical reassessment was sometimes deferred if information could be obtained indirectly, e.g. from the digital patient chart. Physically demanding procedures - such as placing central venous catheters - were more likely to be postponed. One participant explained that when exhausted, they *weigh differently how urgent practical tasks are* (P17), describing a higher threshold for initiating labour-intensive interventions.

Several physicians reported adopting more cautious, conservative decision tendencies when fatigued. One described being *less willing to take risks* and favouring options with low potential for deterioration or subsequent workload (P10). Another participant stated: *If I’m already tired*,* I’m more likely to choose the approach that won’t trigger a cascade of further decisions* (P13). Maintaining the status quo was frequently preferred over proactive intervention when fatigue was pronounced.

And if I ought to do something hands on, despite being completely beat, tired, exhausted, I can’t concentrate anymore, then I weigh up the urgency differently. (P17)

And when you’re already over your limit, you tend to go for the safer option - or the one that involves fewer decisions - simply because you no longer have the capacity for anything else. (P13)

Decisions requiring substantial deliberation or prolonged execution were often deferred due to DF. Participants stated that, deciding whether to do certain tasks was not avoided due to responsibility, but simply because they represented *a lot of work* (P11). This was particularly evident in dealing with awake, demanding or agitated patients, which was described as more challenging when fatigued. In such cases, decisions were postponed or delegated to others, especially towards the end of shifts.

No one really wants to take responsibility for it because it’s a lot of work. I think many people don’t even see it as a matter of responsibility; they just see it as creating a lot of extra work for whoever does it. (P11)

### Low perceived urgency and consequences

When inaction carried no obvious short-term harm, DF was described as more likely to influence decision-making. For example, deferring ventilator weaning by several hours - or even a day - was perceived as clinically acceptable because it was *not time-critical* (P7). Minor diagnostic or therapeutic adjustments were often delayed in a similar way when consequences were diffuse, uncertain or long-term. In contrast, when the consequences of inaction were severe (e.g., risk of cardiac arrest), the influence of DF was commonly described as negligible.

Yes, weaning someone off the ventilator is a process. And it’s not time-critical, it can easily take hours. Whether you reduce the oxygen supply three hours later, or lower the PEEP a bit later, is nothing that has to happen minute by minute, or even within the hour. (P11)

Decisions perceived as non-urgent were frequently reported as showing lower priority when DF was present. Participants described small, accumulating non-urgent requests as *stressful and annoying* when there was *simply no nerve left for them (P2).* Such tasks were more likely to be postponed, handled superficially, or delegated. Their perceived insignificance, combined with mental fatigue, made even minor decisions feel disproportionately effortful, increasing the tendency to avoid or minimise engagement with them.

That really stressed me out. And when all those little crap tasks come at you from every side - from other physicians, from the nurses - it gets even more stressful, because you just don’t have the nerves for it anymore, especially when none of it is actually urgent. (P2)

### Tasks perceived as unpleasant

Finally, decisions associated with tasks viewed as irritating, bureaucratic or burdensome were particularly susceptible to DF. Participants reported forgetting and/or deferring documentation, administrative tasks or tedious follow-up actions when fatigued. Emotional aversion therefore interacted strongly with fatigue with DF being more pronounced when tasks were perceived as unpleasant, reinforcing avoidance behaviours.

When I’m tired, I have no energy for bureaucratic stuff… it feels even more burdensome, so I postpone it, forget it or pass it on to someone else (P19).

## Discussion

### Decision-making as a core professional identity

Our findings show that participants hold a generally positive attitude toward clinical decision-making. Decision processes are perceived as a central component of intensivists’ professional role and are described as expressions of medical expertise, responsibility, and sources of professional meaning and identity.

At the same time, physicians reported that decision-making becomes increasingly burdensome when cognitive and physical resources are depleted. This was particularly evident after demanding workdays and in the context of an accumulation of numerous small, often administrative or repetitive decisions, which were frequently perceived as lacking meaningfulness, disruptive, or detached from direct patient benefit.

Beyond a potential decline in decision quality over time, this process may also compromise job satisfaction and staff well-being. Previous studies have shown that experiencing meaningfulness in one’s work not only predicts job satisfaction^20^ but also buffers the negative effects of work-related stress on overall life satisfaction^21^. When this sense of meaningfulness is subjectively diminished, staff well-being may consequently be at risk.

Therefore, establishing and maintaining conditions that enable clinicians to focus their cognitive resources on clinically meaningful decisions appears to be relevant not only for patient safety but also as a key determinant of staff well-being and the long-term sustainability of professional practice in intensive care medicine.

### Impairments and errors in decision making and team dynamics under DF

In our study, participants perceived DF in intensive care practice as a progressive depletion of cognitive resources, which they perceived to be associated with reduced attention, slower information processing, and less precise execution of essential cognitive tasks. These findings align with existing research and can be interpreted in light of Baumeister’s principles of self-control^4^, although our data do not allow conclusions about underlying cognitive mechanisms.

Participants described decisions made in this depleted state as *more impulsive*,* less deliberate*,* and more driven by gut feeling*, which may be understood in the context of dual-process theories of judgement. According to Kahneman, decision-making operates through two interacting systems: System 1, which is fast, intuitive, and heuristic-based allowing rapid pattern recognition and System 2, which is slower, analytic, and deliberate, but cognitively demanding^22^. In this context, heuristics refer to simplified cognitive strategies or rules of thumb that reduce mental effort during decision-making^23^. Subsequent work has expanded this framework, including Thompson et al.’s emphasis on metacognitive monitoring and the role of control processes^24^, as well as Evans et al.’s proposal to conceptualise the systems in terms of different types of cognitive processes^25^ while maintaining the core distinction between automatic and controlled processing. Beyond dual-process models, Cognitive Continuum Theory^26^ conceptualises decision-making as a dynamic continuum between intuitive and analytical modes, depending on task characteristics such as complexity and structure. This perspective aligns with our findings that different types of clinical decisions may invite different modes of cognition and vary in their susceptibility to fatigue-related influences.

Behaviours reported by participants such as avoidance, decision delay, reduced empathy, and declines in communication quality may also reflect a greater reliance on System 1 under cognitive depletion. Such a shift may represent an adaptive strategy to conserve mental effort; while it can increase susceptibility to certain cognitive biases^27^, intuitive and heuristic-based processing may also be advantageous in familiar or time-critical clinical situations requiring rapid pattern recognition.

Previous studies have demonstrated that the relative contribution of each system is influenced by situational factors^28^. For example, Finucane et al. showed that under time pressure, individuals rely more heavily on emotional components of judgement, thereby favouring System 1 processing^29^. Participants’ descriptions suggest that DF may similarly shift the balance toward System 1 reliance.

These findings can also be interpreted through more recent theoretical perspectives on cognitive and motivational effort. From the standpoint of the self-regulatory model of resource scarcity^6^, physicians’ descriptions of narrowing attention and prioritising immediate or low-effort actions may reflect an adaptive response to a perceived limitation of available cognitive resources. Similarly, within the opportunity-cost framework^7^, participants’ tendencies to defer decisions or select less effortful options can be viewed as signalling that continued engagement was experienced as offering insufficient perceived reward relative to alternative tasks or needs. From the perspective of the opportunity-cost model of mental effort, participants’ frustration with numerous minor administrative or low-impact decisions may reflect a perceived imbalance between effort and reward—these decisions demand cognitive energy while offering little intrinsic or extrinsic reinforcement. Both frameworks align with participants’ accounts of shifting priorities and mental conservation strategies as fatigue accumulated.

Participants perceived this shift as potentially contributing to increased vulnerability to errors, particularly in relation to omission bias, status quo bias, and reduced information processing capacity. Avoidance or postponement of decisions -often through deferral to subsequent shifts- was also described. However, these effects reflect participants’ perceptions rather than objectively measured outcomes.

These cognitive and decisional changes were perceived by participants as potentially relevant for patient afety, including medication-related errors and incomplete information processing. However, these represent hypothesized mechanisms rather than demonstrated causal effects within the present study. Together, these findings highlight that DF is perceived by clinicians as a relevant factor for patient safety in intensive care settings and underline the importance of introducing mitigation strategies. Examples include increasing awareness of DF and cognitive biases, strategically scheduling cognitively demanding tasks at the start of shifts or immediately after breaks^30^, and improving task planning through the establishment of clear team roles, responsibilities, assigned tasks, and backup plans, thereby reducing stress and the need for ad hoc decision-making if the primary plan fails^31^. Empowering nursing staff and junior doctors to make certain decisions autonomously may not only decrease the individual decision load, but should be implemented carefully, as all professional groups are also susceptible to fatigue-related cognitive constraints^32^. Also importantly, participants’ accounts sometimes did not clearly distinguish between decision fatigue and general physical or mental fatigue, suggesting an experiential overlap between these constructs in clinical practice.

### A typology of susceptibility to DF

Our findings suggest that DF does not act as a global or uniform influence but instead disproportionately affects specific types of decisions. Participants indicated that certain decision characteristics may either protect against or amplify the effects of DF. This more differentiated perspective offers a conceptual and hypothesis-generating lens for existing quantitative research and may help explain why some studies demonstrate an influence of DF on medical decision making, whereas others do not.

The low vulnerability of emergency decisions was consistently described by participants and is consistent with prior research showing that such decisions are typically guided by algorithms and embedded within well-structured workflows—conditions that participants described as stabilising and protective. In a study by Stecker et al. examining stroke alert activations, no association was found between potential fatigue and either thrombolytic administration rates or diagnostic accuracy. The authors concluded that clinical performance in this context remains stable despite prolonged work periods, supporting the notion that high-stakes emergencies characterised by clear procedures, high salience, and immediate consequences may be relatively resilient to fatigue-related influences. However, our data reflect perceived resilience rather than objective immunity to DF.

A similar pattern emerges in ethically significant and high-consequence decisions. Interviewees described these decisions as being distributed across team members, revisited over time, and shaped by collective filtering of relevant information. As illustrated in the Results section, participants emphasised the collaborative nature of such ethically weighty decisions: ‘[When making decisions with serious consequences, it’s important] that they are distributed and well justified…’ (P8). Such structural and social buffering mechanisms are likely to contribute to their relative stability under conditions of fatigue. Consistent with this interpretation, studies on teamwork and shared clinical accountability in intensive care have shown that structured team processes and collective decision-making can maintain performance stability even under high workload or fatigue. Coordinated communication, clearly distributed responsibilities, and repeated review of critical decisions appear to buffer the cognitive impact of fatigue and workload, supporting the notion that collaborative structures may protect decision quality in high-stakes contexts^33,34^. Decisions of this kind involve substantial ethical responsibility and quality standards, which may provide an additional protective layer against DF.

In contrast, our results indicate that other types of decisions are particularly susceptible to DF. The decision characteristics identified by participants as vulnerable show similarities to patterns described in prior studies examining workload, fatigue, or performance variation, although these studies do not directly measure decision fatigue as conceptualised here. Hand hygiene compliance, for example, has been shown to decline over the course of a typical 12-hour shift^35^, reflecting tasks with limited immediacy or perceptible consequences for patients and therefore greater susceptibility under fatigue. Similarly, response times to physiological monitor alarms slow with each successive hour of a nurse’s shift, likely reflecting cumulative physical and mental fatigue^36^. In a telephone helpline setting, the likelihood of conservative management decisions increased with each consecutive call taken since the last rest break^37^. The tendency to avoid cognitively demanding or aversive tasks is further supported by studies on radiology reporting, where decreasing report similarity over workdays and workweeks suggests deteriorating report quality under fatigue, particularly among residents^38^.

**Fig. 1:**
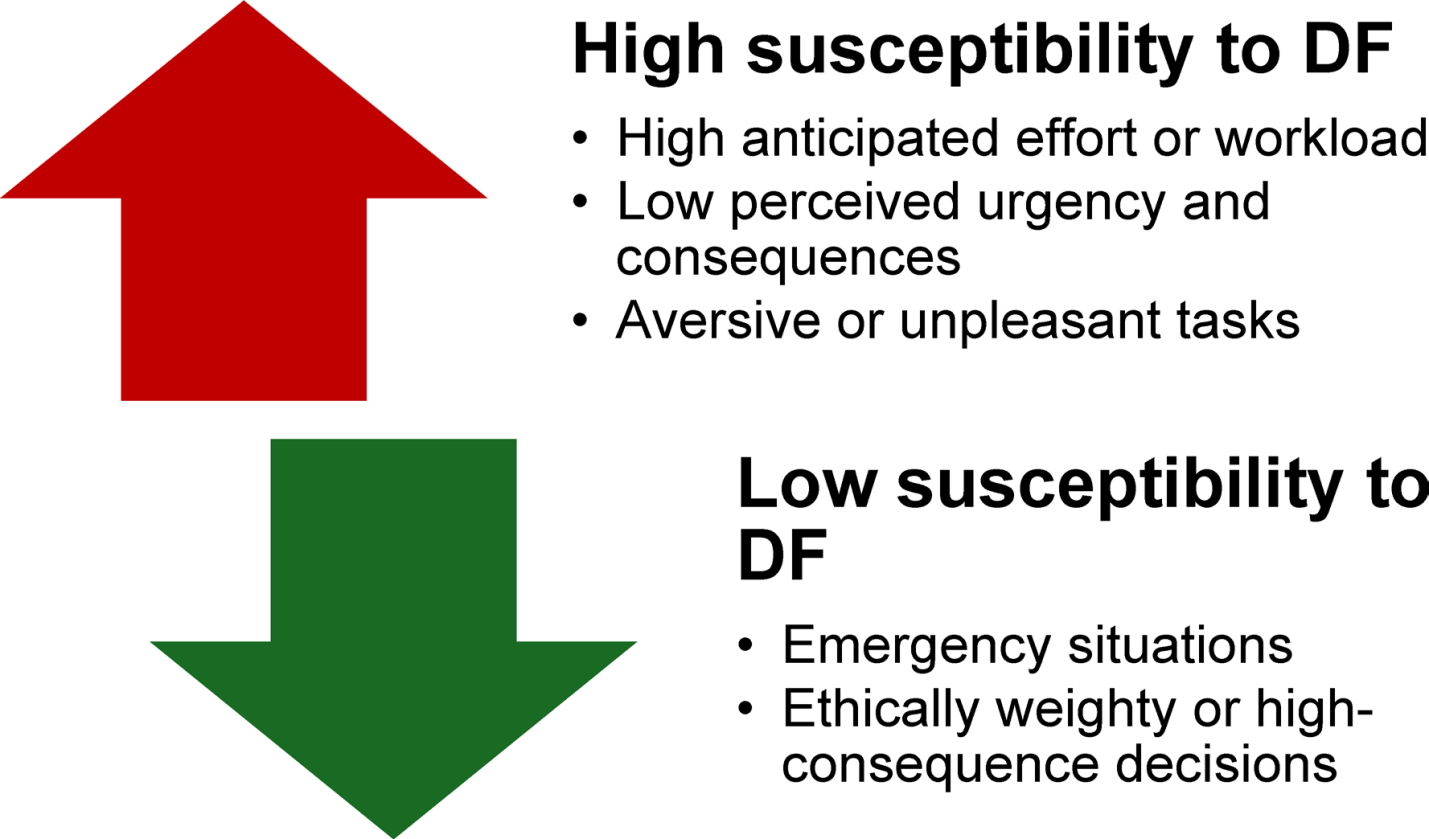
Visualization of clinical decision features participants believed to either increase or decrease susceptibility to DF.

Figure 1 illustrates our typology of clinical decisions according to their perceived susceptibility to DF, based on key decision characteristics identified in our interviews. The typology is intended as a qualitative, hypothesis-generating framework rather than a validated classification.

These findings have several practical implications. Where feasible, cognitively demanding decisions should be prioritised earlier in shifts, when resources are less depleted. Workflows ought to be examined with a view to reducing low-value, repetitive decisions that were consistently described as disproportionately draining. Raising awareness of cognitive biases and heuristic reasoning—through teaching or structured debriefing—may help clinicians recognise fatigue-related shifts in their decision-making. Finally, distributing cognitive responsibility across the team may buffer individual vulnerability to DF, though it should be noted that all professional groups are susceptible to fatigue-related constraints, and redistribution does not eliminate DF but may simply shift it elsewhere. The typology presented here may serve as a practical framework to identify which decisions warrant particular structural protection.

### Strengths and limitations of the study

This study provides a detailed insight into intensivists’ experiences of DF. Several limitations should be noted: it was conducted at a single ICU centre, relied on self-reported interview data, and did not include direct observation of clinical decision-making or outcome measures. The sample was also predominantly composed of residents, and potential differences between professional roles were not the primary focus of this analysis.

In addition, recruitment within a single department may have introduced context-specific organisational influences, and responses may have been affected by social desirability bias and hierarchical dynamics, even though participation was voluntary, and confidentiality was ensured. Furthermore, it is not possible to clearly disentangle DF from related constructs such as general fatigue, workload, sleep deprivation, burnout, moral distress, or cognitive overload. Participants accounts could therefore sometimes reflect a broader experiential overlap between cognitive depletion related to decision-making and general work-related exhaustion.

Despite these limitations, the findings provide valuable hypotheses for future research and potential directions for intervention development. They may guide future research in the selection of decisions under investigation. Rather than treating DF as a generalized state, classifying concrete ICU decisions according to the characteristics identified here may help identify those most susceptible to perceived fatigue-related effects. Such a typology should be considered exploratory and hypothesis-generating, with future studies required to validate and operationalize its components.

## Conclusion

Clinical decision-making constitutes a core element of intensivists’ professional identity and a key source of meaning, yet it becomes increasingly burdensome under cumulative cognitive and physical depletion, with potential consequences for both staff well-being and the sustainability of intensive care practice. When DF manifests it was perceived by participants as being associated with deteriorations in communication and team dynamics and become a risk factor for patient safety.

Our findings indicate that DF does not exert a uniform effect across all clinical decisions but was perceived to affect particularly those characterised by low immediacy, limited perceived consequences, or high cognitive or emotional aversiveness more strongly, as reported by participants. In contrast, urgent, high-stakes, and ethically salient decisions embedded in structured workflows were perceived as relatively resilient due to procedural, social, and contextual buffering mechanisms. The typology presented in this study gives a hypothesis-generating framework, which may help inform future research and targeted strategies to support clinical decision-making in the ICU, including prioritising demanding decisions earlier in shifts, reducing low-value decision load, and strengthening team-based processes.

## Data Availability

The datasets generated and/or analysed during the current study are not publicly available due to participant confidentiality but are available from the corresponding author on reasonable request.

## Abbreviations

DF: Decision fatigue
ICU: Intensive care unit
